# On the way to becoming like anyone else. The experiences of being involved in their care - an interview study with adult patients with ADHD

**DOI:** 10.1080/28324765.2025.2550308

**Published:** 2025-08-22

**Authors:** Petra Becker, A. Birgitta Gunnarsson, Mikael Rask, Jalal Safipour

**Affiliations:** a Department of Research and Development, Region Kronoberg, Växjö, Sweden; b Department of Health and Caring Sciences, Faculty of Health and Life Sciences, Linnaeus University, Växjö, Sweden; c Institute of Neuroscience and Physiology, Department of Health and Rehabilitation, The Sahlgrenska Academy, University of Gothenburg, Gothenburg, Sweden

**Keywords:** Interviews, mental health, neuropsychiatric disorders, nursing, qualitative content analysis

## Abstract

Adults with ADHD face many challenges in their daily lives. Daily life can be made easier by learning from others or developing one’s own strategies. The healthcare services can provide information and interventions, but we know little about what the adults with ADHD themselves say they need, and in which ways they are involved in their care. The purpose of this study is to explore the experiences adults with ADHD have of being involved in their care. Fifteen semi structured in-depth interviews were conducted. The data were analyzed with qualitative latent content analysis that revealed one major theme: “Becoming someone like anyone else” and three sub-themes “Being connected”, “Finding a place in companionship” and “Pathways to participation in care”. The findings of this study emphasize that adults with ADHD express a desire to be involved in the care and treatment for their ADHD if a sense of security and their information needs are met. This involvement becomes part of what leads them towards a more self-sufficient state where they can feel like anyone else and gain more strategies to manage their daily lives.

## Introduction

This study focusses on adults with Attention Deficit Hyperactivity Disorder (ADHD), and their experiences of the care they receive to be able to manage daily life. The causes of ADHD vary from individual to individual, but studies show that in up to 60–80% there is a genetic influence (Faraone & Larsson, 2019). The main symptoms of which are inattention, lack of concentration, impulsiveness, restlessness, and hyperactivity. These symptoms have to have debuted before the age of 12 and be present in multiple settings (e.g. school and home). For adults they also have to be persistent and cause difficulties in adulthood (International Classification of Diseases 11th Revision (ICD-11) (2024). The symptoms of ADHD can manifest themselves in everyday life as difficulties remembering, for example, times, where the individual puts his/her things, but also difficulties starting and completing tasks and getting these tasks carried out in an efficient and appropriate manner. It can also mean difficulties maintaining relationships over time. This could also affect how individuals with ADHD are more likely to experience difficulties adhering to medication plans and consistently taking their medication. Furthermore, additional problems with sleeping, and/or the consequences of comorbidity with mental illnesses and/or substance use disorders are common complications associated with ADHD, which can exacerbate the difficulties (Friedrichs et al., 2012; Sobanski, 2006; Wilens et al., 2002; Zwennes & Loth, 2019). The ability to manage ordinary daily life routines can generally be considered to be an essential part of every person’s life, but is, however, not a reality for everyone. Most adults manage their daily life without any support from healthcare services, but there are those who are in need of care to adapt their habits and routines, and to develop new strategies in daily life (Holst & Thorell, 2020; Toner et al., 2006). These include adults with ADHD who experience difficulties with attention, concentration, impulsivity and hyperactivity, and who might find it difficult to organize, plan and complete tasks (Arellano-Virto et al., 2021; Bjerrum et al., 2017). It has been shown in a recent review (Becker et al., 2023) that adults with ADHD use a variety of selfcare strategies to facilitate daily life, such as gathering information about how the diagnosis can manifest itself, about what help is available from the healthcare and social services, reducing distractions when trying to perform different tasks, and using different aids such as aids for time perception, mobile apps for reminders or calendars or finding helping and supportive relationships (Becker et al., 2023). However, we do not know whether these types of selfcare strategies are sufficient or not, and if they need any additional support from healthcare professionals. Furthermore, there is a lack of knowledge about how sufficient support should be designed, and in this study, we aim to highlight this need from the perspective of the adults with ADHD themselves. The healthcare system in Sweden has the assignment to provide interventions to prevent the occurrence of more serious illness and to facilitate the conditions that exist (SFS, 2017, p. 30). The care and treatment given to adults with ADHD can e.g. involve patient education and prescription of aids or medication (National Program for the Care of and Service for people with ADHD, 2024). Patient education often includes information about the diagnosis, and how to deal with the symptoms in daily life. It can also include non-medical treatment methods such as relaxation exercises or Mindfulness training. Information about various interventions provided by the healthcare and social services, such as various financial benefits or, help at home are often included, and how to gain assistance from the patient associations, such as Attention. The focus of patient education is on increasing people’s ability to manage their daily lives more independently. Medication for reducing the main symptoms of ADHD: hyperactivity, impulsiveness and difficulties in focusing and concentrating, is the most common treatment in Sweden (Polyzoi et al., 2018), but many adults with ADHD tend to stop taking their medication. It was shown in a recent study that only haft of the adults remained on medication during the first year of medication initiation and after five years the number had dropped further (Brikell et al., 2024). Discontinuing medication is considered to be related to adverse effects, treatment ineffectiveness/suboptimal response, dosing inconvenience, and/or social stigma associated with ADHD and/or other psychiatric disorders (Gajria et al., 2014). However, there is a strong evidence for positive effects of the medical treatment (De Crescenzo et al., 2017) and for how non-pharmacological interventions can help adult patients with ADHD (Poissant et al., 2019; De Crescenzo et al., 2017), but to our knowledge there is limited research on which interventions the patients themselves consider to be the most important. Our intention is to explore the experiences of the patients by interviewing those with knowledge of the interventions provided and thus gain a deeper insight into their views of those interventions and of the care encompassing the interventions.

Patients need to know what is available and what can be done in order to obtain the greatest benefit from the care provided. The healthcare services in Sweden have to abide by the regulations in the Patient Act (SFS 2014:821), where it is emphasized that all care given, as far as possible, has to be carried out in mutual agreement with the patient. We have chosen to use the term involved in this study when talking about the collaboration between the patient and their healthcare professional, as this is the term used by the patients themselves (Jerofke-Owen et al., 2023). That means that involvement is the active participation of individuals in decisions and actions related to their treatment and healthcare management. In order for adults with ADHD to become involved in their own care, they should be asked about which type of support and care they desire to receive from the healthcare services so that they can be able to manage their ordinary daily life routines (Jerofke-Owen et al., 2023). There is, to our knowledge, limited research into what adult patients with ADHD consider to be helpful and meaningful care interventions. Such knowledge could further develop and improve the care for adults with ADHD. The aim of this study was thus to explore how adults with ADHD experience being involved in their care.

## Methods

### Design

This study was conducted with an inductive qualitative approach (Polit et al., 2021) as this was considered best for addressing the aim of the study and the nature of the participants’ own experiences, which they presented them during the interviews. Qualitative interviewing (Polit et al., 2021) is a research method that can be used to gather in-depth information about the experiences of individuals through open-ended questions focusing on the understanding and insight within a specific context.

### Context and participants

The participants were recruited from three primary healthcare clinics with responsibility for the care of adults diagnosed with ADHD in southern Sweden. Two of the clinics treated patients with either ADHD or both ADHD and other psychiatric diagnoses, while the third clinic was a specialist clinic specializing in treating adults with an ADHD diagnosis. The inclusion criteria for participation in the study were; 18 years or older, diagnosed with ADHD according to ICD11 (2024), and having a previous or current experience of medical treatment for ADHD. The exclusion criteria were: ongoing addiction, i.e., alcohol or other drugs, or severe difficulties in communicating in the Swedish language and thus unable to participate in a meaningful interview. All the participants had ADHD as their main diagnosis, while many of them spoke of having other diagnoses, e.g., depression, anxiety and autism.

A designated registered nurse (RN) from each clinic was assigned to ask patients who met the inclusion criteria about their participation in the study. Those who were interested were given a pamphlet with short information about the study. Contact details were forwarded to the first author (PB) when a patient consented to participate. All of the patients had received both oral and written information by the time of the interview, and all of them signed an informed consent to participate in the study prior to starting the interview. The recruitment of participants was ongoing until it could be perceived as having sufficient depth and variety.

Seventeen adults met the inclusion criteria and were asked to participate in the study. Two of them declined at a later date due to other illnesses. Eight of the remaining 15 participants were women and seven were men. Their ages ranged between 20 and 55 years and they had been diagnosed with ADHD for nine months to 15 years, with a median of 2.5 years (Table 1). All participants were diagnosed with ADHD in adulthood. The selection of participants from different locations, the equal distribution between female/male participants, and the variation in age and length of time since being diagnosed was considered to contribute to both depth and variation in the collected data.

**Table 1.** Study participants*

Length of time since ADHD diagnosis	Age	Female	Male
0 to 1 year			
	20	Cecilia	
	21	Beatrice	
	52		Axel
	37	Anna	
1 to 5 years			
	21		Clas
	25		Adam
	30	Anita	
	36		Anton
	37	Bodil	
	46	Carina	
	55		Bengt
5 years and above			
	27	Barbro	
	31	Camilla	
	41		Carl
	47		Birger

*All names are fictional and correspond to the citations provided in the results.

### Data collection

The data were collected in semi structured in-depth interviews, lasting from 30 to 75 minutes. The interviews were performed face-to-face (*n* = 12) or in Zoom meetings (*n* = 3). This latter option was added due to the covid-19 pandemic. The participants were given the opportunity to choose where they wanted the interview to take place. The face-to-face interviews were all conducted where the participants usually received their care. All the interviews, which were conducted by the first author (PB) and audio recorded, were carried out between August 2020 and August 2022. The main reason for the long timespan was the covid-19 pandemic and the lockdown that took place in Sweden, where restrictions on meeting unknown people were limited by the government. Five interviews were conducted prior to the lockdown and ten afterwards. All the interviews were transcribed verbatim.

### Interviews

An interview guide (Table 2), which was developed by the research team prior to the data collection, was partly based on major themes found in a previous literature review (Becker et al., 2023). This review identified three themes that facilitate daily life for adults with ADHD; “*Establishing ways of acting to help yourself*”, “*Finding encouraging and helping relationships*”, and “*Using external aids for managing daily life*” (Becker et al., 2023). All the interviews, which were conducted by the first author, started with the participants being asked to talk about what made them suspect they might have had ADHD, and why they turned to the healthcare services for support. These participant narratives were further explored with follow-up questions based on the given theme of the interview guide. The participants were requested to share experiences of their participation in their care, e.g. experiences of care given and received, and whether they experienced any lack of care or not. Furthermore, the interviews dealt with their experiences of engagement, motivation and participation in care and about the interventions and medical treatment that they have received. The participants were asked to clarify, elaborate and provide examples to better describe their experiences if the interviewer experienced any ambiguity or uncertainty in the participants’ narratives.

**Table 2.** Interview guide

The interview guide consists of a number of open-ended questions. The informant is given the opportunity to deepen or clarify the answers during the interview with the help of follow-up questions. The purpose of these follow-up questions is to obtain further information about the phenomenon being studied.
Tell us what it was like for you to be diagnosed with ADHD. Tell us how you experience your care. Which treatment have you received? Tell us about your experiences of these treatments. Is there anything that you feel you are missing in your care? Do you feel that you are involved in your care? Can you give examples of when you have been involved/when you have not been involved? Tell us what could contribute to you experiencing greater participation. Have you ever discontinued treatment? If so, can you tell us why you discontinued your previous treatment? Is there anything that would prevent you from making the decision to discontinue your care? Give some examples.

### Ethical considerations

Ethical approval was granted by the Swedish Ethical Review Authority (Dnr 2019-06381). All participants received written and oral information about the study before the interviews took place. Participation was voluntary and they received information that they could withdraw at any time. All participants provided written informed consent before study enrollment according to the Declaration of Helsinki (2013).

### Data analysis

The data were analyzed with qualitative content analysis (Graneheim & Lundman, 2004). The interviews were transcribed verbatim either by the first author or by a professional transcriber before starting the analysis. All identification marks in the transcriptions were removed. The data were read through by all the authors, and several times by the first author (PB). Meaning units, i.e. words or sentences that responded to the aim of this study, were marked and picked out. These were then condensed and coded. The first author (PB) coded all the data, and the other authors (ABG, MR & JS) participated in the coding of at least two interviews each. All codes were compared in order to arrive at a common interpretation. When the coding was completed, all of the codes were compared in order to seek out similar content and patterns among the codes in search of a common interpretation of subthemes and themes (Table 3), in accordance with Lindgren et al. (2020). This process continued until consensus was reached.

**Table 3.** Example of analysis from meaning unit to theme

Meaning unit	Condensed meaning unit Description close to the text	Condensed meaning unit Interpretation of the underlying meaning	Sub-theme	Theme
Mmm. X and I have talked a lot, so he’s always been a person, I´ve always been a person, I´ve been able to turn him and ask him, because we’ve become very close and … it´s about trust there, mutual trust then…(Adam)	Being a person, I could turn to and ask questions, be close to, it’s about trust and reciprocity.	To feel that there is a person you trust and can turn to	Being connected	Becoming someone like anyone else
It was quite a lot in the beginning, then it was … when I started with medication it was very often, back then we met once a month and had very deep discussions and tried out medications. And then … we met maybe once a month in one year. (Adam)	In the beginning we met often, regularly and had deep discussions, and then we met maybe once a month.	Being able to meet		
But in any case … then that doctor said anyway that… when I came and said that, that yes, but he has tried… now I don’t remember … Ritalin and all these different medications, and it gave him such a huge headache and this one he didn’t want … he didn´t like, but then this Elvanse then has been spot on from the lowest dosage to the highest dosage he had././No, but then she said, I can confirm that, she said, because it´s also about the feeling that she was also controlled in her own way, I then understood, like first we have to try this and then we can try this and last we can try this one././Yeah, the last one has to be the best was the thought one got … laughter … (Bengt)	Have tried different preparations but suggested Elvanse to find one that suited and was confirmed by the doctor in this	To feel listened to for your experiences	Pathways to participation in care	
I was offered to attend this ADHD-program, but I haven´t been able to do this unfortunately, because it´s interfered with … I mean, I have my kids at preschool and stuff, but I only have them for fifteen hours././and it’s so inconvenient times like this, so I can’t seem to get away from it, so I would like them to change, that they had more opportunities, so you could actually go, because they said that several people had complained about that.(Bodil)	Was offered ADHD school, but the times didn’t suit and I wish they would adapt more	To be given the conditions to be able to participate		

All the authors have previous experience of using qualitative methods in data analysis. Three of them have extensive experience working in mental healthcare, two of whom are nurses, and one is an occupational therapist. The fourth author works in the field of medical science.

## Results

The main theme “*Becoming someone like anyone else*” expresses the experiences that contribute to the participants´ involvement in their care and treatment in order to be better able to manage their daily life activities. Their openness to receive care generates opportunities for them to become like anyone else, thus helping them be able to live their lives as other people do. One of the motivational drives for involvement in care for patients with ADHD concerns feeling secure and safe in daily life activities and being connected to others such as relatives, friends and healthcare professionals. Involvement in care and treatment contributes to personal growth that generates feelings of empowerment and satisfaction with care intervention for patients with ADHD. Ultimately, this involvement allows them to carve out their own space in society and establish a secure foundation for independence. Three subthemes describe the areas that interact to create these feelings of safety, belonging and security, “*Being connected*”, “*Finding a place in companionship*” and “*Pathways to participation in care*”.

### Being connected

The knowledge of where and to whom to turn to in healthcare situations was described as creating a sense of security by most of the participants. The existence of continuity in the contact with the same healthcare professional over time helped to make the participants feel secure with both their healthcare plans and the healthcare professional. To be able to have a designated healthcare professional to reach out to when needed was described as an elementary foundation for creating this sense of security in the relationship. This was felt because that person knew their story and there was no need to repeat the same information each time, even if there were intervals between visits;*” more like my nurse, who makes me feel secure, I don´t have to explain over and over again about my difficulties, because she knows”*. (Camilla, 31 years old). We use ´contact person` in the rest of the manuscript as the term for the member of staff who is the designated healthcare professional for each patient.

The participants talked of the importance of knowing that the healthcare professionals were accessible when they sought contact, but also that it was easy to get in touch with one´s contact person. This could also be expressed by the participants as being cared for in such a way that the care was understandable and not full of bad experiences. Difficulties in contacting or maintaining contact were instead described as reasons for lowering the participants’ levels of engagement in their own care.

The participants described having feelings of loss, abandonment or frustration when they were left alone, with no or insufficient information about how or where to turn to get in touch with their healthcare units or contact person*” … but I’ve ended up being a bit in between…one of them thinks I should do something and the other says something else. And then I don’t know what to do at all”*. (Anita, 30 years old)

The participants also described the importance of the contact person staying in touch with them. They experienced it as difficult to make contact, to remember to stay in touch and to maintain contact. Having an understanding with one´s contact person that this was a mutual responsibility was experienced as a relief.

*… if I cancel my appointment I’ll be contacted again…I know, she’ll call me. I know she’ll call me and hear how it’s been and … and it’s been great because then I know, yeah but I always have someone who comes back to me.*

(Bodil, 37 years old)

### Finding a place in companionship

The participants said that to be able to be involved in their care it was important to be part of a larger context where they felt at home and were recognized, a sense of companionship. Facilitating a connection between patients in order to foster support and create a sense of community around shared life experiences, was identified as a crucial responsibility for healthcare professionals. Creating the circumstances to find a place where others with similar difficulties were and where they had the opportunity to share coping strategies, and in return gain knowledge of other ways to cope was an engaging experience.

*… it was a group who all had an ADHD diagnosis, and each one had one or two relatives accompanying them. And it was great fun, because that was really the point, that okay, I’m not alone in this, you knew that before too, but it was a room in which I saw normal people, but who had the same diagnosis as myself … it made a big impression*

(Adam, 25 years old)

Meeting people with similar experiences also gave them role models outside of the healthcare services with whom they felt a sense of companionship. Conversations with others in a similar situation, being able to feel that one could contribute with one’s experiences and create a community both for relatives and for other adults with ADHD were described as strengthening one’s self-esteem.

*There you could actually discuss setbacks and yes everything that has to do with life with ADHD, with other people… And just the fact that the relatives got to join in and talk to other relatives who are going through the same struggles and share their experiences … .*

(Carl, 41 years old)

Recognizing oneself in other people’s descriptions and gaining insight that one was not alone in needing help and support from healthcare professionals or others was described as giving layers of strength, a sense of belonging, and of being part of a context. Seeking affinity with others in similar situations in order to be able to share the burden was experienced as helpful, not only for the participants themselves but also for their relatives. Group activities, such as free family ADHD courses for patients and their relatives, were described as both supportive and informative, and were seen to strengthen and sustain their bond with other patients, family members and healthcare professionals.

### Pathways to participation in care

The participants talked of taking great responsibility in being involved in their care and hoped this engagement would enable them to make their daily lives more manageable. They described the importance of the healthcare professionals inviting and encouraging them to become involved in their care when being given the opportunity to influence the decisions regarding their care. The participants experienced it as being meaningful when the staff listened to what they had to say about their life experiences and they themselves were allowed to make suggestions for treatment. It was also important that the healthcare professionals responded with openness and provided the conditions for the participants to enter into the conversation, to feel they were seen and listened to, so that they dared to say what they wanted. Experiencing good communication and being able to discuss and reach solutions regarding their care was said to be decisive for the experience of participation”; *There have been some discussions with… we may not be quite there yet, about which medicine is the best, but then… they have been open for discussions, so they have, and they listen when I say something”* (Axel, 54 years old).

The participants highlighted some areas as being more important in order to be able to become involved, e.g., information and knowledge, everyday conversations that lead to a good and safe relationship with their contact person, and being recognized as a person not just one with difficulties but one with substantial experience of the situation.

A number of factors were identified by the study participants as being important in order to be able to choose what the participants thought suited them best or in which manner the care should be given. These included obtaining information and knowledge about opportunities for various educations, support given by the healthcare services but also in the community and also aids such as applications, clocks or calendars or references to web pages or written information about the diagnosis or medicines and medical treatment.

The participants had experiences of receiving information that they recognized as being trustworthy. This was often information that was adapted to the recipient and given both verbally and in writing. On the other hand, getting too little information or getting information about things they already knew or had experience of was not good. They spoke of seeking knowledge about the ADHD diagnosis independently on the internet, but also gained knowledge by way of diagnostic educational courses.

*… so, I haven’t received much information about that, like when I switched to Concerta, I got leaflets home with me about what it is and what it means and so on… but most of the information I had to research myself, I joined a group for ADHD instead (edit note - group on Facebook)*

(Beatrice, 21 years old)

The participants had experience of taking computer-based educational courses at home but found it difficult to be focused and assimilate the course content this way.

*… but in this particular format, I think it’s a bit difficult, it’s hard to keep your concentration when you have to sit at the computer and listen to it, I think it gets boring and a little…you get stressed by sitting at home and watching so, because you know you can do something more important.*

(Clas, 21 years old)

Ordinary conversations about situations in daily life, with its problems, and also conversations in the psychoeducational context about the diagnosis and the effect of the medication were spoken of. The participants stated that when they had contact with a healthcare professional they could trust, they often turned to this person for advice and support about both major and minor matters. Conversations about daily life, how different situations could arise and how to deal with them in a constructive way provided guidance in managing daily life more easily”; *… in other words, providing a structure for doing some cleaning and things like that, household chores, and helping me apportion my energy for the whole day, so that I can cope and so on…”* (Bodil, 37 years old)

The participants highlighted the importance of feedback and being confirmed by one´s contact person when having done well even if everything was not perfect, was particularly important during these conversations about daily life. Relatives were also highlighted by the participants as important, both in terms of support in making good decisions, and as a help to remember what was said during visits to the healthcare services.

The participants talked of a number of experiences that could lead to them feeling they were not invited and not having the opportunity to become engaged and then losing interest in participating. These experiences included: not being confirmed or listened to, meetings with the healthcare services that were experienced as lacking opportunities to have influence, the healthcare professionals only being influenced by their own guidelines, and feelings of being questioned, disregarded or excluded. They also spoke of trying to participate, making decisions where they did not feel they had sufficient knowledge as being overwhelming for them and that they were given too much responsibility without any feeling of having somewhere to turn for help.

*… but at the same time, I didn’t get to make the decision myself, because they had given rules…, yes, we do it first, we always try this way at this end. I might have wanted to skip these steps and move on to something else. They gave information about all the medicines, but at the same time I didn’t really get to choose.*

(Carina, 46 years old)

## Discussion

This study explores the experiences of adult patients, diagnosed with ADHD, related to their engagement and involvement in their care and treatment. The main findings were that the participants, by becoming engaged and involved aspired to “*Becoming someone like anyone else”*. This meant that their desire to be accepted by others and to fit into the community, i.e., “living life like other people”, motivated them. Not being like others was a feeling that was never far away and finding solutions to more easily manage their daily lives was therefore of great value.

The importance of having a safe and prescribed connection to a healthcare facility and to one’s contact person was highlighted in the subtheme *being connected*. The participants described comparably simple things, such as knowing where and to whom to turn when needed, as being important. Continuity in the relationship with one´s contact person over time was one of the elements in creating the security and safety that the participants spoke of. They felt that it was necessary in order for the care to be beneficial and for feeling able to be involved and engaged in one´s care. This is in line with a study by Hamovitch et al. (2018), who found that “connection, continuity and calibration” are the basis of the therapeutic relationship for both participants and providers, especially when designing a person-centered care plan (PCCP).

Furthermore, the therapeutic relationship has been shown to not only influence the patient’s engagement but also the outcome of the patient’s care (Mallonee et al., 2022). Patients who are involved in their care get more out of it, e.g. concerning symptom reduction, improvement in global functioning and greater satisfaction with treatment (Hamovitch et al., 2018; Kidd et al., 2017).

It was revealed in this study that the participants wanted to feel safe and cared for by the healthcare professionals, and that if the healthcare professionals were committed then the participants were also committed. On the other hand, they lost confidence in the care when the support from the healthcare professionals was insufficient, or there was no continuity. It is described in a study by Meaux et al. (2009) that when healthcare professionals do not engage or are not committed in the meetings with adults with ADHD, the meetings tend to be superficial and the person does not take the opportunity to ask questions or become engaged in their care.

Being a patient, living with ADHD, places different demands on the care relationship in comparison with a patient being treated for a simple ailment, e.g., a sore throat or a cough. The patient with ADHD expects and requests a long-term relationship with their healthcare professional, a therapeutic relationship that is based on trust and equality between them (Hedman, 2020). The continuity of such a therapeutic relationship is usually described as important as the patients do not have to tell their life story over and over, and instead can talk about crucial situations in their present daily life. Denhov and Topor (2012) also describe interpersonal continuity in terms of a relationship to one person or a team. This relationship is the basis for making patients feel safe and being able to be a part of their care.

The sub-theme “*Finding a place in companionship*” describes the participants being able to share their experiences through their companionship with others, but while also gaining an insight into the fact that they were not alone. This was experienced as giving hope to find ways to a daily life in the future that works for them. These findings could be related to those in a study by Björk et al. (2021), which concluded that adults with ADHD perceived confidence and could encourage each other through trusting relationships with other participants in the study, which in turn increased their motivation to carry out the intervention. Groups for psychoeducation organized within healthcare service are a way of providing an opportunity for adults with ADHD to meet. In this study these groups are described by the participants as a meeting place for themselves and for their relatives. Moreover, Skliarova et al. (2024) found in a recent review that psychoeducation as an intervention showed significant improvements concerning, e.g. ADHD symptoms over time.

Different opportunities for openings to become involved and engaged in care were described by the participants in the sub-theme “*pathways to participation in care”*. Communicating knowledge, i.e., information to facilitate involvement, the necessity of involving relatives, and having a contact person who listens and understands their daily struggles was revealed to be important This has also been shown by Larsson et al. (2007), who highlighted the nurse-patient interaction, the necessity of having appropriate, credible and sufficient information, and of the importance of considering the patient as the person in focus.

The findings of this study also revealed the importance of being given the right kind and amount of information in different ways; information about the diagnosis in general and the consequences for daily life that the symptoms have, and about possible options for care and treatment. The information was experienced as being helpful when addressed directly towards adults with ADHD in order to help them be able to understand, engage and make appropriate decisions concerning planning for their care and treatment. By providing such information and supporting the patient to make their own decisions, the healthcare professional assisted the patient towards increased empowerment. This is in line with Hickmann et al. (2022), who maintains that by increasing the patient’s empowerment, e.g. the ability to think critically, and independently make decisions that benefit their own health will increase the patient’s willingness and ability to become involved in their own care.

The findings of this study showed that the participants with ADHD shared experiences about the importance of a stable care-contact over time in which they could feel that there was a trusting and safe alliance being developed. They were able to contact their healthcare professionals and receive the help and support that was specifically adjusted to their needs when the relationship was stable. This led to them being empowered and becoming more engaged in their care. Previous studies (Hamann et al., 2003; Krist et al., 2017) indicate that patients who are involved in their care and take a more active role are more satisfied with their care, have more knowledge about their diagnosis and potential treatment strategies, and have more realistic expectations about what they can receive in terms of medications and other support interventions. Moreover, studies have also shown that adherence to medication has improved when the patient is allowed to participate in the decisions regarding their treatment (Kripalani et al., 2007; Matthes & Albus, 2014).

It is crucial when patients perceive that they can be involved in their care to make it comprehensible by encouraging them to be a more active partner in order for their care to be more beneficial. Different types of structured patient participation exist, such as shared decision-making, patient-centered care or person-centered care (Hickmann et al., 2022). Healthcare professionals can assist patients to take a more active role, and thus influence their own care and treatment by using these different types of structured models or decision aids. These can lead to patients gaining greater knowledge of their options, feeling more informed and secure about their decisions and more satisfied with the relationships with healthcare professionals (Entwistle et al., 2014; Kirsch & Matthes, 2021). The findings of this study emphasize that if the needs of adults with ADHD about feelings of safety and information are met, then they expressed a desire to want to be involved in their care and treatments in terms of how to cope with their symptoms from ADHD. This can become a part of what can lead them towards a more self-sufficient existence where they can feel like anyone else and take control of their lives.

## Methodological considerations

This study has strengths and weaknesses and our considerations of these are based on the terms pertaining to trustworthiness as proposed by Graneheim and Lundman (2004).

The credibility (Graneheim & Lundman, 2004) was strengthened by using a qualitative design (Polit et al., 2021), conducting in-depth interviews with adults who were diagnosed with ADHD, with the aim of exploring how they experienced their involvement in their care given by healthcare professionals. The credibility was further strengthening by using the themes about selfcare strategies to cope with daily life (Becker et al., 2023) in the development of the interview guide.

Although the data material from the interviews with the participants in this study were seen to be of a similar nature, the selection of the participants was based on the inclusion criteria and a variation in terms of age, gender, duration of diagnosis, and experiences of care was obtained, which also strengthened the credibility (Graneheim & Lundman, 2004).

Excluding individuals who could not communicate in Swedish may have limited the diversity of perspectives in our sample, particularly in terms of immigrant populations. This decision was made to ensure the quality and consistency of the data, as the interviews required nuanced understanding and expression of personal experiences.

The COVID-19 pandemic led to the need of having to adapt to special conditions and challenges when carrying out interviews. This meant that not all interviews could be conducted face-to-face, some of them were instead conducted via zoom meetings. Not being able to meet in a room may have affected the interviews, for example, all the non-verbal communication is perhaps not transmitted via the computer screen in a zoom-meeting in the same way as in a face-to-face interview. However, we maintain that it was important that the same interviewer (PB) conducted all the interviews, and that questions of uncertainty that needed clarification were perceived and could be addressed during the interviews, which in turn strengthened the dependability, in line with Graneheim and Lundman (2004).

The study has been analyzed with qualitative latent content analysis, which is suitable for the analysis of the participants’ experiences of being involved in their care, and is a research method well known to the researchers. There is strength in having a prior understanding of the subject under study, but it is also important to strengthen the dependability of interpretation when categories and subcategories are formed. This was carried out by all the researchers participating in the analysis and performing the different steps in the analysis independently and then together in order to gain a consistency in the findings (Graneheim et al., 2017).

Three of the researchers in this study have extensive clinical experience (PB, MR, ABG) and experience of both research and work with patients within different psychiatric care and treatment contexts. One researcher (JS) has experience of working in the field of mental health. The dependability of the study was strengthened by all the researchers participating with their knowledge of the research method, the analysis and clinical experience of the studied subject (Graneheim & Lundman, 2004).

The findings presented in this study are supported by previous research and are in line with the importance of the relationship between patients and their healthcare professionals (Hamovitch et al., 2018; Mallonee et al., 2022), and what stimulates patients to become more involved and engaged in their care (Entwistle et al., 2014; Kirsch & Matthes, 2021). The potential transfer of the findings of this study to other healthcare facilities needs to be carefully considered as there may well be differing contextual factors in, for example, other psychiatric healthcare facilities. However, the findings in this study are similar to those in other studies, and the possibilities for transferring the findings to adult patients with ADHD in other settings is thus strengthened.

## Conclusion

The participants in this study had both positive and negative experiences of being involved in their care. Their main goal for engaging in care was to become more like others. Finding themselves, accepting and embracing the help offered, and becoming an active participant in their care were indicated as constituting important elements in being able to make their daily lives feel more as a whole. When one of the parts failed, the emphasis was on balancing the other parts to increase the sense of involvement and commitment. Nurses and healthcare professionals can play an essential role in facilitating the participants to become involved and to continue to be involved. How the care is structured in terms of availability and continuity also emerged as decisive for the participants to continue their commitment.

The experiences of the participants’ involvement in their care and treatments have been explored in this study, but a major element in a person’s life is their relatives, family and friends and the need to support each other. To gain a greater understanding of the needs for support of adults with ADHD it is thus also necessary to explore the experiences of their relatives.

## Relevance for clinical practice

The findings in this study have potential to increase awareness among healthcare professionals about the benefits of having a participatory approach when engaging patients in their healthcare planning. It shows the importance of person-centered care and the therapeutic relationship between the patient and healthcare professionals in order to stimulate the patients’ involvement and strengthen their own ability to deal with difficulties in daily life. This study provides increased knowledge for decision-makers when developing new guidelines for care and treatment of adults with ADHD by highlighting the areas that increase the opportunities for patients to be able to participate actively in their care.

## References

[cit0001] Polit, D. F., & Beck, C. T. (2021). *Essentials of Nursing research: Appraising evidence for Nursing practice* (10th ed.). Wolters Kluwer.

[cit0002] Arellano-Virto, P. T., Seubert-Ravelo, A. N., Prieto-Corona, B., Witt-González, A., & Yáñez-Téllez, G. (2021). Association between psychiatric symptoms and executive function in adults with attention deficit hyperactivity disorder. *Psychology and Neuroscience*, 14(4), 438–453. 10.1037/pne0000271

[cit0003] Becker, P., Rask, M., Safipour, J., & Gunnarsson, A. B. (2023). Selfcare strategies shown to Be useful in daily life for adults diagnosed with attention deficit hyperactivity disorder - a systematic review. *Issues in Mental Health Nursing*, 44(9), 1–9. 10.1080/01612840.2023.2234477 37669505

[cit0004] Bjerrum, M. B., Pedersen, P. U., & Larsen, P. (2017). Living with symptoms of attention deficit hyperactivity disorder in adulthood: A systematic review of qualitative evidence. *JBI Database of Systematic Reviews and Implementation Reports*, 15(4), 1080–1153. 10.11124/JBISRIR-2017-003357 28398986

[cit0005] Björk, A., Rönngren, Y., Hellzén, O., & Wall, E. D. (2021). The importance of belonging to a context: A nurse-led lifestyle intervention for adult persons with ADHD. *Issues in Mental Health Nursing*, 42(3), 216–226. 10.1080/01612840.2020.1793247 32809885

[cit0006] Brikell, I., Yao, H., Li, L., Astrup, A., Gao, L., Gillies, M., Xie, T., Zhang-James, Y., Dalsgaard, S., Engeland, A., Faraone, S., Haavik, J., Hartman, C., Ip, P., Jakobsdóttir Smári, U., Larsson, H., Man, K., de Oliveira Costa, J. Pearson, S.& Klungsøyr, K. (2024). ADHD medication discontinuation and persistence across the lifespan: A retrospective observational study using population-based databases. *Lancet Psychiatry*, 11(1), 16–26. 10.1016/S2215-0366(23)00332-2 38035876

[cit0007] Declaration of Helsinki. (2013). World medical association de claration of Helsinki: Ethical principles for medical research involving human subjects. https://www.wma.net/what-we-do/medical-ethics/declaration-of-helsinki/10.1001/jama.2013.281053 24141714

[cit0008] De Crescenzo, F., Cortese, S., Adamo, N., & Janiri, L. (2017). Pharmacological and non-pharmacological treatment of adults with ADHD: A meta-review. *Evidence-Based Mental Health*, 20(1), 4–11. 10.1136/eb-2016-102415 27993933 PMC10699262

[cit0009] Denhov, A., & Topor, A. (2012). The components of helping relationships with professionals in psychiatry: Users’ perspective. *International Journal of Social Psychiatry*, 58(4), 417–424. 10.1177/0020764011406811 21602221

[cit0010] Entwistle, V. A., Brown, R. C. H., Morgan, H. M., & Skea, Z. C. (2014). Involving patients in their care. *Current Breast Cancer Reports*, 6(3), 211–218. 10.1007/s12609-014-0151-2

[cit0011] Faraone, S. V., & Larsson, H. (2019). Genetics of attention deficit hyperactivity disorder. *Molecular Psychiatry*, 24(4), 562–575. 10.1038/s41380-018-0070-0 29892054 PMC6477889

[cit0012] Friedrichs, B., Igl, W., Larsson, H., & Larsson, J. O. (2012). Coexisting psychiatric problems and stressful life events in adults with symptoms of ADHD-A large Swedish population-based study of Twins. *Journal of Attention Disorders*, 16(1), 13–22. 10.1177/1087054710376909 20686099

[cit0013] Gajria, K., Lu, M., Sikirica, V., Greven, P., Zhong, Y., Qin, P., & Xie, J. (2014). Adherence, persistence, and medication discontinuation in patients with attention-deficit/hyperactivity disorder - a systematic literature review [article]. *Neuropsychiatric disease and treatment, vol 2014, iss default*, (pp. 1543–1569) (*2014*)(default), 1543. http://search.ebscohost.com/login.aspx?direct=true&db=edsdoj&AN=edsdoj.fe1a7fa4667a48d3a87804364848b145&lang=sv&site=eds-live 10.2147/NDT.S65721 PMC4149449 25187718

[cit0014] Graneheim, U. H., Lindgren, B.-M., & Lundman, B. (2017). Methodological challenges in qualitative content analysis: A discussion paper. *Nurse Education Today*, 56(Supplement C), 29–34. 10.1016/j.nedt.2017.06.002 28651100

[cit0015] Graneheim, U. H., & Lundman, B. (2004). Qualitative content analysis in nursing research: Concepts, procedures and measures to achieve trustworthiness. *Nurse Education Today*, 24(2), 105–112. 10.1016/j.nedt.2003.10.001 14769454

[cit0016] SFS 2017: 30. (2017) Hälso- och sjukvårdslagen. https://www.riksdagen.se/sv/dokument-och-lagar/dokument/svensk-forfattningssamling/halso-och-sjukvardslag-201730_sfs-2017-30/

[cit0017] Hamann, J., Leucht, S., & Kissling, W. (2003). Shared decision making in psychiatry. *Acta Psychiatrica Scandinavica*, 107(6), 403–409. 10.1034/j.1600-0447.2003.00130.x 12752015

[cit0018] Hamovitch, E. K., Choy-Brown, M., & Stanhope, V. (2018). Person-centered care and the therapeutic alliance. *Community Ment Health J*, 54(7), 951–958. 10.1007/s10597-018-0295-z 29948627

[cit0019] Hedman, H. (2020). Patientens - personens röst. In I. R Ekman (Ed.), *Personcentrering inom hälso- och sjukvård : från filosofi till praktik* (p. 362 sidor). Liber AB.

[cit0020] Hickmann, E., Richter, P., & Schlieter, H. (2022). All together now - patient engagement, patient empowerment, and associated terms in personal healthcare. *BMC Health Services Research*, 22(1). 10.1186/s12913-022-08501-5 PMC9440506 36056354

[cit0021] Holst, Y., & Thorell, L. (2020). Functional impairments among adults with ADHD: A comparison with adults with other psychiatric disorders and links to executive deficits. *Applied neuropsychology-Adult*, 27(3), 243–255. 10.1080/23279095.2018.1532429 30624968

[cit0022] International classification of diseases 11th revision (ICD-11). (2024) WHO, World Health Organization. https://www.who.int/standards/classifications/classification-of-diseases

[cit0023] Jerofke-Owen, T. A., Tobiano, G., & Eldh, A. C. (2023). Patient engagement, involvement, or participation - entrapping concepts in nurse-patient interactions: A critical discussion. *Nursing Inquiry*, 30(1), 1–9. 10.1111/nin.12513 35871476

[cit0024] Kidd, S. A., Davidson, L., & McKenzie, K. (2017). Common factors in community Mental Health intervention: A scoping review. *Community Mental Health Journal*, 53(6), 627–637. 10.1007/s10597-017-0117-8 28194599

[cit0025] Kirsch, V., & Matthes, J. (2021). Aspects of medication and patient participation-an easy guideLine (AMPEL). A conversation guide increases patients’ and physicians’ satisfaction with prescription talks. *Naunyn-Schmiedeberg’s Archives of Pharmacology*, 394(8), 1757–1767. 10.1007/s00210-021-02107-0 34106304 PMC8298249

[cit0026] Kripalani, S., Yao, X., & Haynes, R. B. (2007). Interventions to enhance medication adherence in chronic medical conditions: A systematic review. *Archives of Internal Medicine*, 167(6), 540–550. 10.1001/archinte.167.6.540 17389285

[cit0027] Krist, A. H., Tong, S. T., Aycock, R. A., & Longo, D. R. (2017). Engaging patients in decision-making and behavior change to promote prevention. *Studies in Health Technology and Informatics*, 240, 283–302. 10.3233/978-1-61499-790-0-284 PMC6996004 28972524

[cit0028] Larsson, I. E., Sahlsten, M. J., Sjöström, B., Lindencrona, C. S., & Plos, K. A. (2007). Patient participation in nursing care from a patient perspective: A grounded theory study. *Scandinavian Journal of Caring Sciences*, 21(3), 313. 10.1111/j.1471-6712.2007.00471.x 17727543

[cit0029] Lindgren, B.-M., Lundman, B., & Graneheim, U. H. (2020). Abstraction and interpretation during the qualitative content analysis process. *International Journal of Nursing Studies*, 108, 103632. 10.1016/j.ijnurstu.2020.103632 32505813

[cit0030] Mallonee, J., Petros, R., & Solomon, P. (2022). Explaining engagement in outpatient therapy among adults with serious mental health conditions by degree of therapeutic alliance, therapist empathy, and perceived coercion. *Psychiatric Rehabilitation Journal*, 45(1), 79–88. 10.1037/prj0000490 34138609

[cit0031] Matthes, J., & Albus, C. (2014). Improving adherence with medication: A selective literature review based on the example of hypertension treatment. *Deutsches Arzteblatt international* (Vol. 111, pp. 41–47). 24612495 10.3238/arztebl.2014.0041 PMC3950822

[cit0032] Meaux, J. B., Green, A., & Broussard, L. (2009). ADHD in the college student: A block in the road. *Journal of Psychiatric & Mental Health Nursing*, 16(3), 248–256. 10.1111/j.1365-2850.2008.01349.x 19291153

[cit0033] National Program for the Care of and Service for people with ADHD. (2024). *Sveriges kommuner och regioner (SKR)*. Retrived from https://www.vardochinsats.se/adhd/

[cit0034] Poissant, H., Mendrek, A., Talbot, N., Khoury, B., & Nolan, J. (2019). Behavioral and cognitive impacts of mindfulness-based interventions on adults with attention-deficit hyperactivity disorder: A systematic review. *Behavioural Neurology*, 2019, 5682050. 2019. 10.1155/2019/5682050 31093302 PMC6476147

[cit0035] Polyzoi, M., Ahnemark, E., Medin, E., & Ginsberg, Y. (2018). Estimated prevalence and incidence of diagnosed ADHD and health care utilization in adults in Sweden - a longitudinal population-based register study [artikel]. *Neuropsychiatric Disease and Treatment*, 14, 1149–1161. 10.2147/NDT.S155838 29765219 PMC5944447

[cit0036] SFS 2014: 821, *The patient act*. (2014). Department of Health and Social Affairs. https://www.riksdagen.se/sv/dokument-och-lagar/dokument/svensk-forfattningssamling/patientlag-2014821_sfs-2014-821/

[cit0037] Skliarova, T., Pedersen, H., Holsbrekken, Å., Pedersen, S. A., Mandal, A., De Las Cuevas, C., Havnen, A., Gråwe, R., & Lara-Cabrera, M. L. (2024). Psychoeducational group interventions for adults diagnosed with attention-deficit/hyperactivity disorder: A scoping review of feasibility, acceptability, and outcome measures. *BMC psychiatry*, *24*(1), 463. 10.1186/s12888-024-05908-8 PMC11191191 38902683

[cit0038] Sobanski, E. (2006). Psychiatric comorbidity in adults with attention-deficit/hyperactivity disorder (ADHD). *European Archives of Psychiatry and Clinical Neuroscience*, 256, i26–i31. 10.1007/s00406-006-1004-4 16977548

[cit0039] Toner, M., O’Donoghue, T., & Houghton, S. (2006). Living in chaos and striving for control: How adults with attention deficit hyperactivity disorder deal with their disorder. *International Journal of Disability, Development and Education*, 53(2), 247–261. 10.1080/10349120600716190

[cit0040] Wilens, T. E., Spencer, T. J., & Biederman, J. (2002). A review of the pharmacotherapy of adults with attention-deficit/hyperactivity disorder. *Journal of Attention Disorders*, 5(4), 189–202. 10.1177/108705470100500401 11967475

[cit0041] Zwennes, C. T. C., & Loth, C. A. (2019). “Moments of failure”: Coping with attention deficit hyperactivity disorder, sleep deprivation, and being overweight: A qualitative hermeneutic-phenomenological investigation into participant perspectives. *Journal of Addictions Nursing*, 30(3), 185–192. 10.1097/JAN.0000000000000291 31478966

